# Pharmacological Inhibition of PKCθ Counteracts Muscle Disease in a Mouse Model of Duchenne Muscular Dystrophy

**DOI:** 10.1016/j.ebiom.2017.01.001

**Published:** 2017-01-07

**Authors:** V. Marrocco, P. Fiore, A. Benedetti, S. Pisu, E. Rizzuto, A. Musarò, L. Madaro, B. Lozanoska-Ochser, M. Bouché

**Affiliations:** Dept. of Anatomy, Histology, Forensic Medicine and Orthopedics, Unit of Histology and Med. Embryology, CE-BEMM and Interuniversity Institute of Myology, Sapienza University of Rome, 00161 Rome, Italy

**Keywords:** ANP, Atrial Natriuretic Peptide, C20, Compound 20, ConA, Concanavalin A, CSA, cross sectional area, CsA, Cyclosporin A, DGC, dystrophin-glycoprotein complex, DIA, Diaphragm, DMD, Duchenne Muscular Dystrophy, DMSO, Dimethyl Sulfoxide, DTT, Dithiothreitol, EDL, extensor digitorum longus, EDTA, Ethylenediaminetetraacetic acid, EGTA, ethylene glycol-bis(β-aminoethyl ether)-*N*,*N*,*N*′’,*N*′’-tetraacetic acid, eMyHC, embryonic Myosin Heavy Chain, GA, Gastrocnemius, IgG, Immunoglobulin G, LV, Left Ventricle, PBS, phosphate buffered saline, PK, Pharmacokinetics, PKCθ, Protein Kinase C-theta, PMSF, phenylmethylsulfonyl fluoride, RT, room temperature, TA, Tibialis Anterior, TGFβ, Transforming growth factor beta, βMyHC, beta-myosin heavy chain, Muscular dystrophy, Protein Kinase C theta, Muscle repair, Inflammation, Therapy

## Abstract

Inflammation plays a considerable role in the progression of Duchenne Muscular Dystrophy (DMD), a severe muscle disease caused by a mutation in the dystrophin gene. We previously showed that genetic ablation of Protein Kinase C θ (PKCθ) in *mdx*, the mouse model of DMD, improves muscle healing and regeneration, preventing massive inflammation. To establish whether pharmacological targeting of PKCθ in DMD can be proposed as a therapeutic option, in this study we treated young *mdx* mice with the PKCθ inhibitor Compound 20 (C20). We show that C20 treatment led to a significant reduction in muscle damage associated with reduced immune cells infiltration, reduced inflammatory pathways activation, and maintained muscle regeneration. Importantly, C20 treatment is efficient in recovering muscle performance in *mdx* mice, by preserving muscle integrity. Together, these results provide proof of principle that pharmacological inhibition of PKCθ in DMD can be considered an attractive strategy to modulate immune response and prevent the progression of the disease.

**Research in context:**

Duchenne muscular dystrophy (DMD) is a severe muscle disease affecting 1:3500 male births. DMD is caused by a mutation in dystrophin gene, coding for a protein required for skeletal and cardiac muscle integrity. Lack of a functional dystrophin is primarily responsible for the muscle eccentric contraction-induced muscle damage, observed in dystrophic muscle. However, inflammation plays a considerable role in the progression of DMD. Glucocorticoids, which have anti-inflammatory properties, are being used to treat DMD with some success; however, long term treatment with these drugs induces muscle atrophy and wasting, outweighing their benefit. The identification of specific targets for anti-inflammatory therapies is one of the ongoing therapeutic options. Although blunting inflammation would not be a “cure” for the disease, the emerging clue is that multiple strategies, addressing different aspects of the pathology, which may eventually converge, may be successful. In this context, we previously showed that genetic ablation of Protein Kinase C θ (PKCθ), an enzyme known to be involved in immune response, in *mdx*, the mouse model of DMD, improves muscle healing and regeneration, preventing massive inflammation. To establish whether pharmacological targeting of PKCθ in DMD can be proposed as a therapeutic option, in this study we treated young *mdx* mice with the PKCθ inhibitor Compound 20 (C20). We show that C20 treatment led to a significant reduction in muscle damage associated with reduced immune cells infiltration, reduced inflammatory pathways activation, and maintained muscle regeneration. Importantly, C20 treatment is efficient in recovering muscle performance in *mdx* mice, by preserving muscle integrity. Together, these results provide proof of principle that pharmacological inhibition of PKCθ in DMD can be considered an attractive strategy to modulate immune response and prevent the progression of the disease.

## Highlights

• Immune-cell intrinsic PKCθ activity might play a hitherto unrecognized role of in the development of DMD.
• *Mdx* dystrophic mice were treated with the PKCθ inhibitor C20.
• C20 treatment prevents damage and inflammation in dystrophic muscle, while improving muscle regeneration.
• C20 treatment prevents drop in force and ameliorates fatigue resistance in dystrophic mice.

## Graphical Abstract

**Figure:**
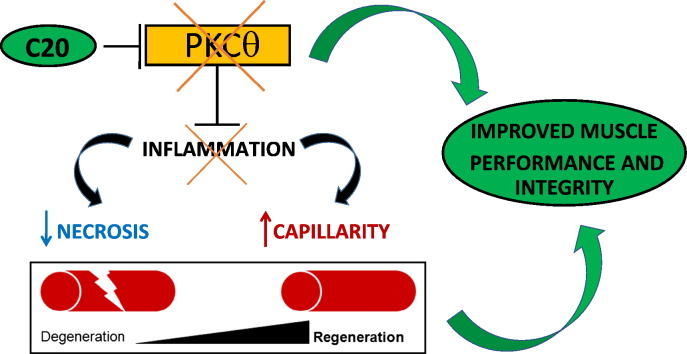

## 1 Introduction

Duchenne Muscular Dystrophy (DMD) is a severe muscle disease affecting 1:3500 male births. DMD is caused by a mutation in dystrophin gene, coding for a protein required for skeletal and cardiac muscle integrity (Mercuri and Muntoni, 2013, Muntoni, 2003, Muntoni et al., 2003). Indeed, dystrophin is an essential component of the dystrophin glycoprotein complex (DGC) which connects the extracellular matrix with the intracellular actin filaments (Muntoni et al., 2003). Lack of a functional dystrophin is primarily responsible for the muscle eccentric contraction-induced muscle damage, observed in dystrophic muscle (Kumar et al., 2004). However, several altered mechanisms are involved in DMD onset and progression, resulting in muscle weakness and progressive muscle wasting (Evans et al., 2009a). Because of muscle damage, cytokines expression is stimulated and immune cells infiltrate in dystrophic muscle, contributing to worsen the dystrophic phenotype (Evans et al., 2009b, Rosenberg et al., 2015, Villalta et al., 2015). Indeed, dystrophic muscles are characterized by up-regulation of inflammatory gene expression, such as NF-kB, and by an increase in inflammatory cell infiltration, mostly composed by macrophages, neutrophils, and T cells. Macrophages, neutrophils, and T cells are the primary cells that invade the dystrophic muscle. It is currently believed that neutrophils and macrophages infiltrate the skeletal muscle around age 2 weeks in mdx mice, while CD8 + and CD4 + T cells infiltrate the muscle between age 4–8 weeks (Evans et al., 2009a, Evans et al., 2010, Rosenberg et al., 2015). NF-kB pathway results persistently activated in dystrophic muscle and it has been proposed as one of the major mediators of muscle wasting: it is activated prior the onset of the disease in response of increased Calcium influx and mechanical stretch and it is responsible for the increased cytokine expression and the immune response activation (Acharyya et al., 2007, Kumar and Boriek, 2003, Messina et al., 2006, Messina et al., 2009). The repeated cycles of myofibers degeneration and regeneration, due to lack of dystrophin, lead to the establishment of a chronic inflammatory environment (Madaro and Bouche, 2014). Thus, the functional muscle tissue is replaced by a fibrotic and adipose non-functional connective tissue (Kharraz et al., 2014, Pessina et al., 2015). Therapeutic strategies based on the restoration of dystrophin expression or the administration of dystrophin^+ ve^ stem cells are promising, but are often limited in efficacy, and long-lasting efficacy has still to be reached (Bengtsson et al., 2016, Cossu et al., 2015, Odom et al., 2011, Sampaolesi et al., 2003, Sampaolesi et al., 2006, Seto et al., 2012). The identification of specific targets for anti-inflammatory therapies is one of the ongoing therapeutic options. Although blunting inflammation would not be a “cure” for the disease, the emerging clue is that multiple strategies, addressing different aspects of the pathology, which may eventually converge, may be successful. Glucocorticoids, which have anti-inflammatory properties, are being used to treat DMD with some success; however, long term treatment with these drugs induces muscle atrophy and wasting, outweighing their benefit (Angelini, 2007, Angelini and Peterle, 2012, Schacke et al., 2002, Schoepe et al., 2006). Numerous other anti-inflammatory therapies, aimed to target more specific mediators of inflammation, have been proposed to improve healing (Heier et al., 2013, Pelosi et al., 2015). In this context, we previously showed that lack of PKCθ in *mdx*, the mouse model of DMD, in the *mdx/*PKCθ^−/−^ mouse model we generated, was associated with reduced muscle wasting, improved muscle regeneration and maintenance of performance compared to *mdx* mice (Madaro et al., 2012). We further demonstrated, by bone marrow transplantation experiments, that PKCθ expression in immune cells is required to mount a robust inflammatory response in *mdx*, which, in turn, exacerbates the muscle pathology (Madaro et al., 2012). PKCθ belongs to the novel class of PKCs and is expressed in both hematopoietic cells and in striated muscle (Marrocco et al., 2014, Nath and Isakov, 2014). In T cells, it is the key member of the PKC family to play a critical role in the Ca2 +/NFAT, AP-1 and NF-kB pathways to activate the IL-2 and IL-4 promoters, and it appears to be required for the development of a robust inflammatory response in vivo (Pfeifhofer et al., 2003, Thuille et al., 2013, Wachowicz et al., 2014, Wachowicz and Baier, 2014, Kwon et al., 2012). Previous studies showed that PKCθ^−/−^ mice fail to develop experimental allergic encephalomyelitis, display drastically reduced lung inflammation after induction of allergic asthma, and have a significantly diminished response in experimental colitis and a type II collagen induced arthritis model (Anderson et al., 2006, Fang et al., 2012, Tan et al., 2006, Wang et al., 2009). Of note, PKCθ^−/−^ mice may still mount a normal protective immune response to clear viral infections (Giannoni et al., 2005), and maintain T_reg_ function (Gupta et al., 2008, Ma et al., 2012, Sun, 2012, Zanin-Zhorov et al., 2010). Taken together, these evidences validate PKCθ as a particularly attractive target to selectively manipulate T_eff_ cell functions that are relevant to pathogenesis of different diseases, including, as our results suggested, muscular dystrophy. Noteworthy, a large effort is now devoted to the synthesis of pharmacological PKCθ inhibitors, to be used for anti-inflammatory therapies, and several PKCθ inhibitors have been identified and reported in recent literature (Boschelli et al., 2010, Chand et al., 2012, Curnock et al., 2014, Cywin et al., 2007, George et al., 2015, Hage-Sleiman et al., 2015, Jimenez et al., 2013, Katoh et al., 2016). Some of them bind selectively PKCθ while others are non-selective in nature. One of the highly selective inhibitor for PKCθ reported was C20, (compound 20 from Boheringer-Inglheim Pharmaceuticals, Inc), belonging to the amino pyrimidine class of inhibitors (Cywin et al., 2007, Sims et al., 2007, Zanin-Zhorov et al., 2010). In any case, to propose a pharmacological protocol to inhibit PKCθ, eventual possible effects on other tissues should be considered. Indeed, PKCθ is expressed also in other tissue, including skeletal and cardiac muscle. In skeletal muscle, we and others showed that PKCθ is mostly involved in mediating signaling pathways regulating fetal and early post-natal growth and maturation (Madaro et al., 2011, Madaro et al., 2013, Marrocco et al., 2014, Messina et al., 2010, Zappelli et al., 1996). Employing PKCθ^−/−^ mice, we also showed that PKCθ maintains the correct structure and function of the heart by preventing cardiomyocyte cell death in response to work demand and to neuro-hormonal signals, to which heart cells are continuously exposed (Paoletti et al., 2010).

Nevertheless, we already showed that genetic ablation of PKCθ in *mdx* does not lead to apparent adverse effects, while it significantly ameliorates the progression of the disease, preventing a robust inflammatory response (Madaro et al., 2012). In this study, we show that in vivo pharmacological inhibition of PKCθ in *mdx* mice significantly ameliorates the dystrophic phenotype, at both morphological and functional levels.

## 2 Materials and Methods

### 2.1 Animal Models

*Mdx* mice (C57BL/10ScSn-Dmdmdx/J) were purchased from Jackson laboratory and *mdx*PKCθ −/− transgenic mice were generated in our laboratory (C57BL/6j-C57BL/10ScSn background). C57BL/10ScSn control mice (WT mice) were purchased from Jackson laboratory. Only males were used. The animals were housed in the Histology Department–accredited animal facility. All the procedures were approved by the Italian Ministry for Health and were conducted according to the U.S. National Institutes of Health (NIH) guidelines.

### 2.2 Compound 20 and In vivo Treatment

**Supplemental Fig. S1:**
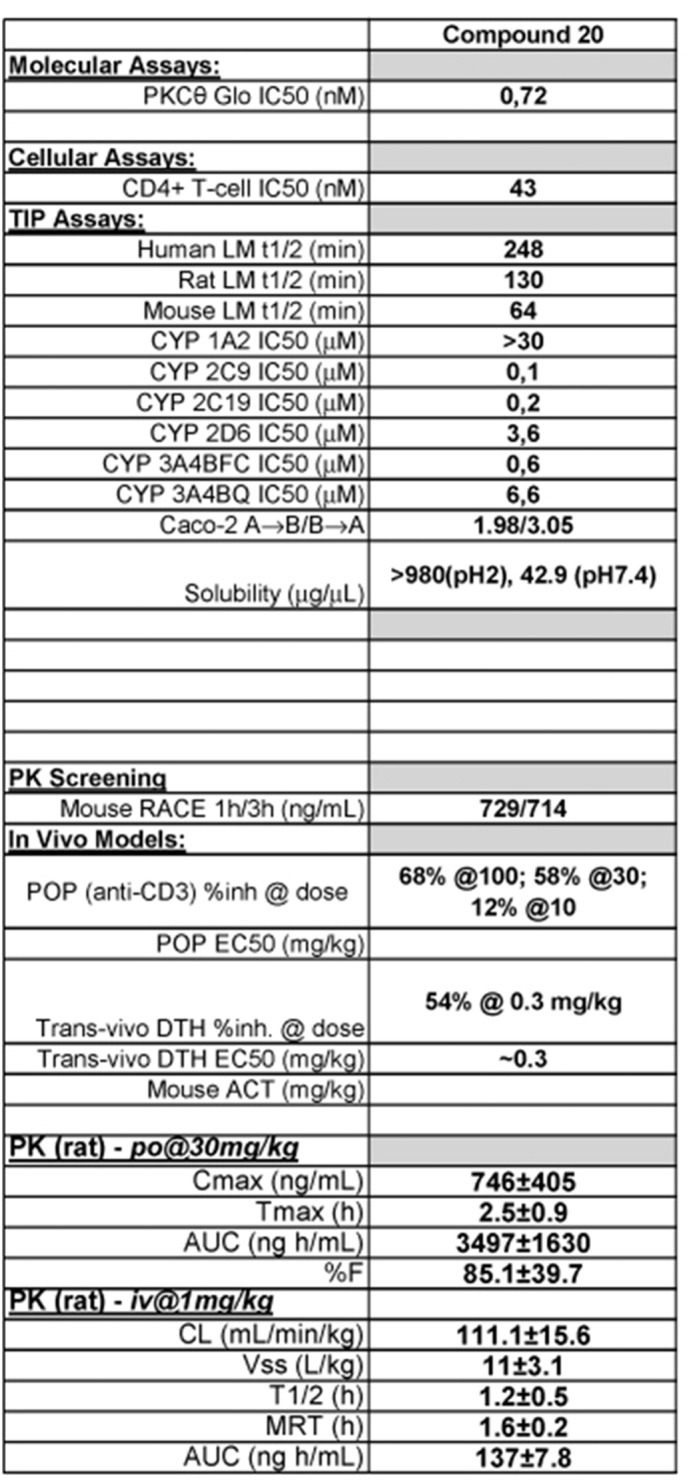
Molecular characteristics and pharmacokinetics (PK) parameters of C20 determined from oral delivery in both mouse and rat models (kindly provided by Dr. M. Brown). RACE: Rapid Assessment of Compound Exposure; Cmax: the maximum concentration recorded; tmax: the time take to reach Cmax; AUC: Area Under the Curve; t1/2: elimination half-life; DTH: Delayed Type Hypersensitivity; Vss: volume in steady state; CL: clearance; MRT: Mean Residence Time; CYP: Cytochrome P450; POP: Proof of Pharmacology.

The PKCθ-specific inhibitor compound 20 (C20) was gently provided by Dr. Maryanne Brown (Boehringer Ingelheim Pharmaceuticals Inc., Ridgefield, CT, USA) (Cywin et al., 2007). Pharmacokinetics (PK) data are summarized in Fig. S1, and show that C20 has a good in vivo activity with oral delivery in T cell mediated models. Oral delivery is usually harder than intra-peritoneal, to get a decent exposure. The rat PK data is complete and the mouse exposure is similar to rat levels at 2.5 h (M. Brown, personal communication)

Unless differently specified, four-week-old mice were daily intra-peritoneally injected with 10 mg/kg C20 or vehicle (10% DMSO is physiological solution) for 2 weeks. At the end of treatment, at age 6 week, the mice were sacrificed and the muscles were analyzed at both molecular and histological level.

To induce muscle atrophy by food deprivation, mice were maintained for 24 h, starting early in the morning the day before being sacrificed, with no food but free access to water (Madaro et al., 2013).

### 2.3 Concanavalin A (ConA) Induced IL-2 Production and ELISA Assay

WT mice were pre-treated, with a single intra-peritoneal (IP) 5 or 10 mg/kg dose of C20, or with the vehicle alone; in parallel, a group of WT mice was pre-treated with 30 mg/kg Cyclosporine A (CsA) (Sigma-Aldrich, Saint Louis, MO, USA), as control. After 1 h following pre-treatment with C20 or CsA, mice were intravenously injected with a single dose of 8 mg/kg ConA (Sigma-Aldrich). In parallel, a group of PKCθ −/− mice was also treated with the ConA or with the vehicle (10% DMSO in physiologic solution). After 2 h, the mice were bled via cardiac puncture and sera were analyzed for IL-2 production by ELISA.

For the IL-2 quantification in serum, a Mouse Interleukin-2 ELISA kit (Thermo Scientific™, Waltham, MA, USA) was used, following the manufacturer's protocol. The kit is very sensitive (< 3 pg/ml) and it has a good reproducibility (intra-assay variability < 10%). The absorbance was measured on a plate reader at 550 nm.

### 2.4 Antibodies

The following antibodies were used: the anti-p-NFκB p65 (Ser536) (clone 9H31) and the anti-NFκB p65 (clone C22B4) from Cell Signaling Technology (Danvers, MA, USA); the anti-Gapdh (clone 6C5) from Santa Cruz Biotechnology (Dallas, Texas, USA); the anti-embryonic Myosin Heavy Chain, eMyHC (clone F1.652) from Developmental Studies Hybridoma Bank (Iowa City, IA, USA); polyclonal anti-Laminin from Sigma-Aldrich; the anti-CD45 PE/Cy5 conjugated (clone 30-F11) from BD Pharmigen™ (San Jose, CA, USA); the anti-CD31 Alexa Fluor 488 conjugated (clone MEC13.3) from Biolegend (San Diego, CA, USA); the anti-mouse IgG TRITC conjugated and the anti-rabbit IgG TRITC conjugated from Sigma Aldrich; the anti-mouse IgG Alexa Fluor 488 conjugated from Invitrogen; the anti-rabbit IgG Chromeo 488 conjugated from Abcam (Cambridge, MA, USA); the anti-mouse IgG and the anti-rabbit IgG HRP conjugated from Bethyl Laboratories (Montgomery, TX, USA).

### 2.5 Western Blotting

For total protein extract preparation, GA muscles derived from vehicle and C20 treated *mdx* mice were homogenized in ice-cold buffer containing 20 mM Tris (pH 7.5), 2 mM EDTA, 2 mM EGTA, 250 mM sucrose, 5 mM DTT, 200 mg/ml leupeptin, 10 mg/ml Trasylol, 1 mM PMSF, and 0.1% Triton X-100 and then disrupted by sonication. The homogenate was incubated for 30 min on ice with repeated vortexing, then centrifuged at 12,000*g* for 10 min at 4 °C. The pellet was discarded. An aliquot of the supernatants was used for protein determination using the Comassie Plus protein assay reagent (Pierce, Rockford, IL), according to the manufacturer's instruction, while the remainder was used for Western blot analysis. An equal amount of protein from each sample was loaded onto 10% SDS-polyacrylamide gel and transferred to a nitrocellulose membrane (Schleicher and Schuell, Dassel, Germany). The membranes were incubated with the appropriate primary and secondary antibodies, and processed as previously described (Madaro et al., 2011). Densitometric analysis was performed using the Image J software (NIH, Bethesda, MA, USA).

### 2.6 Flow Cytometry

For FACS analysis, Gastrocnemius muscles (GA) from *mdx* mice, treated with the vehicle and with the C20, were collected and then digested with Collagenase type IV for 1 h and 30′ at 37 °C with agitation. The cells were passed through a 70 μm and then a 40 μm cell strainer, centrifuged at 1200 rpm and suspended in 100 μl of 1% FBS in PBS (phosphate buffered saline). The cells were stained with the CD45 PE/Cy5 antibody (clone 30-F11 from Pharmigen TM) and with DAPI and then analyzed by the FacsStar Plus cytofluorimeter.

### 2.7 Histological and Immunofluorescence Analyses

Individual limb muscles (Tibialis Anterior, TA, and GA), Diaphragm (DIA) and heart were dissected. Thereafter, cryosections were prepared for histological and immunohistochemical analyses. Sections stained with hematoxylin/eosin or with Masson's trichrome stain (both from Sigma-Aldrich) were photographed and the images were analyzed using Image J. Cross sections of the TA muscle around its mid-portion were used to measure muscle cross sectional area. The total number of myofibers was also counted, as well as the number of myofibers per square millimeter. For immunohistochemistry, crysections were fixed in 4% paraformaldehyde (Sigma-Aldrich) at room temperature (RT) and then incubated with PBS containing 1% BSA for 15 min at RT, before being incubated with PBS containing 5% goat serum for 30 min at RT. The cryosections were then incubated overnight at 4 °C with the appropriate primary antibodies. The day after they were washed with PBS, incubated for 15 min with 1% BSA in PBS, and then incubated for 1 h with fluorochrome-labeled secondary antibodies and Hoechst 33342 (Fluka) for nuclear staining. After extensive washing with PBS, the slides were mounted in 10 mM Tris-HCl, pH 9.00, containing 60% glycerol, and examined in a Zeiss Axioskop 2 Plus fluorescence microscope.

The muscle fiber mean cross sectional area (CSA) was determined by immuno-staining for α-laminin and measuring CSA of fibers in the entire section, using the macro for Image J for automatic fiber CSA assessment. Myofiber variability was determined by multiplying the standard deviation of all measurements by 1000 and dividing it by the mean fiber diameter (Madaro et al., 2012). IgG immune-staining was used to identify damaged myofibers while eMyHC (embryonic Myosin Heavy Chain) was used to identify regenerating myofibers (Madaro et al., 2012). The extent of the area containing IgG- or eMyHC-positive myofibers was measured by Image J in 3 different GA sections/mouse. For each section, we calculated the ratio between positive area and the total area of the section. The average from the three different sections/mouse was calculated and plotted on the graph.

### 2.8 Exhaustion Treadmill Test

The analyses were carried out using a five-lane motorized treadmill (LE 8710, PanLab S.L.U., Barcelona, Spain) supplied with shocker plates. Mice were first acclimated to the treadmill by making them run 20 min at 6 m/min the day before the beginning of the test (at day -1). In the protocol used, the treadmill was run at an inclination of 0° at 8 cm/s for 5 min, after which the speed was increased by 2 cm/s every min. The test was stopped when the mouse remained on the shocker plate for > 20 s without attempting to reengage the treadmill. The time to exhaustion was determined from the beginning of the test. Three tests were performed on the same animal, once per week. All the cohorts run together and all the different groups were done at the same time.

### 2.9 Muscle Functional Studies Ex vivo

Extensor digitorum longus (EDL) muscles were vertically mounted in a temperature controlled (30 °C) chamber containing Krebs Ringer bicarbonate buffer (Sigma K-4002) continuously gassed with a mixture of 95% O_2_ and 5% CO_2_ (pH 7.3 ± 0.3; osmolarity: 267 ± 5% mOsm/L). One end of the muscle was linked to a fixed clamp while the other end was connected to a force transducer (Aurora Scientific Instruments 300B). EDL muscles were electrically stimulated by means of two platinum electrodes with 300 mA controlled current pulses (Del Prete et al., 2008). To evaluate the decline of force, 5 tetanic stimulations were imposed to the muscle with a recovery period of 2 min between each of them (Pelosi et al., 2007). The maximum force was measured from the first tetanic stimulation. The Muscle CSA was calculated dividing the muscle mass by the product of L_f_ and the density of mammalian skeletal muscle (1.06 mg/mm^3^). L_f_ is the optimal fiber length and is obtained by multiplying the muscle length (L_0_) to the fiber length ratio, which is 0.44 for EDL muscle.

### 2.10 RNA Extraction and Quantitative Real Time PCR

Total RNA from muscle tissues was extracted using the TRIsureTM (Bioline, London, UK), converted in cDNA using the High-Capacity cDNA-RT kit from Applied Biosystem, according to supplier's instructions. PCR amplification was performed using the Euroclone FluoCycle™ II SYBR® Green, following the manufacturer's protocol. For data analysis, the 7500 Software v2.0.5, provided by Applied Biosystem, was used. All PCR reactions were carried out in triplicate (duplicate for the standard curves). All qPCR results are expressed as relative ratios of the target cDNA transcripts to β*-actin* and normalized to that of the reference condition. The following primers were used for amplification: ANP-for TGG GAC CCC TCC GAT AGA TC; ANP-rev AGC GAG CAG AGC CCT CAG T; βMyHC-for TCT CCT GCT GTT TCC TTA CT; βMyHC-rev GTA CTC CTC TGC TGA GGC TT; TGFβ-for GGATACCAACTATTGCTTCAGCTCC; TGFβ-rev AGGCTCCAAATATAGGGGCAGGGTC; eMyHC-for TCGTCTCGCTTTGGCAA; eMyHC-rev AGCAGCTCAATCAGCTCA.

### 2.11 Statistical Analysis

Quantitative data are presented as means ± SD of at least three mice, as indicated for each experiment. Statistical analysis to determine significance was performed using paired Student's *t*-tests or with ANOVA test. Differences were considered statistically significant at the p < 0.05 level.

## 3 Results

### 3.1 In vivo C20 Treatment Prevents T Cell Activation but Does Not Alter Skeletal Muscle and Heart Functions

**Fig. 1:**
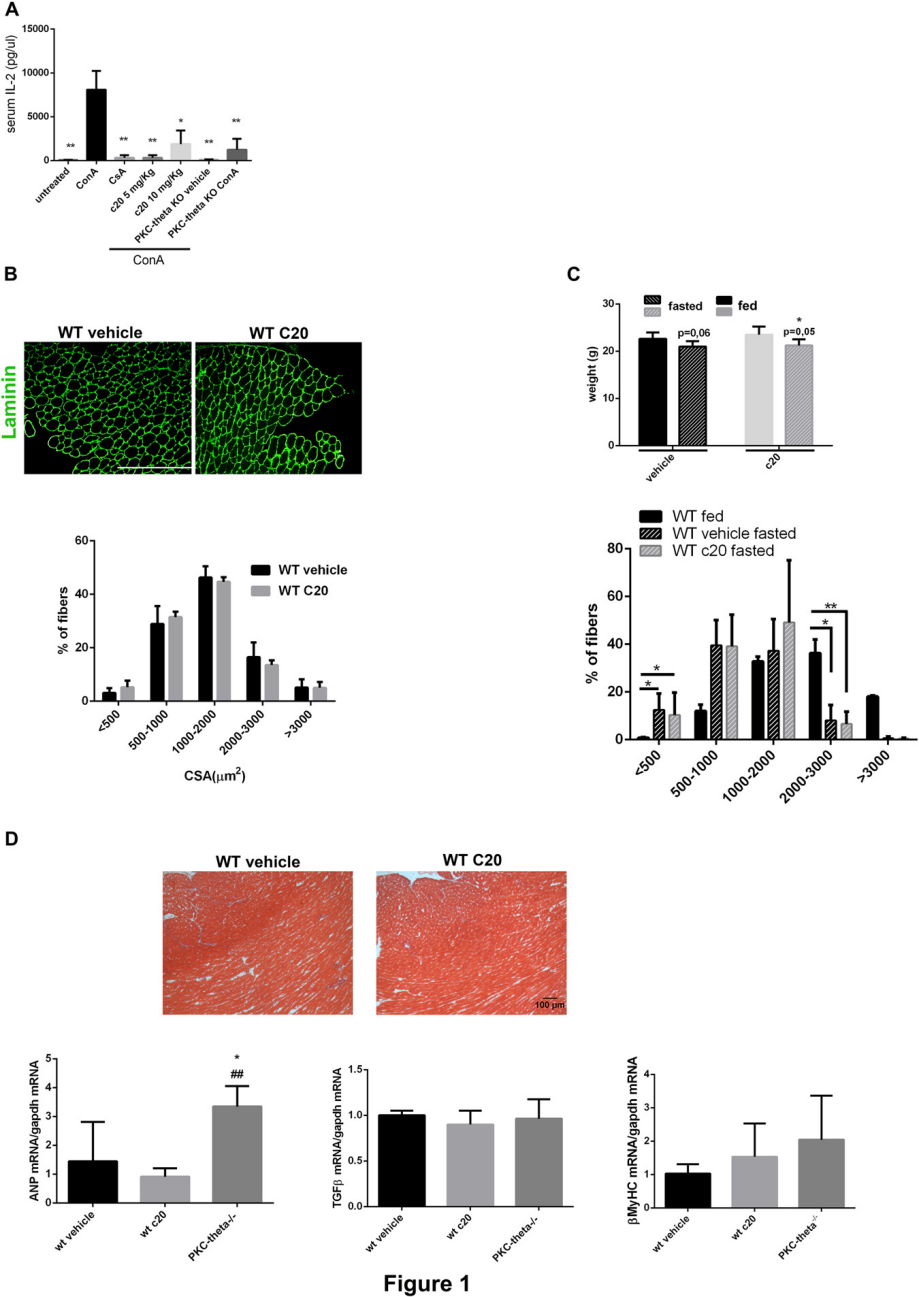
In vivo C20 treatment prevents T cell activation and does not alter skeletal muscle and heart functions. A. ELISA assay of IL-2 serum level in ConA treated WT mice, pre-treated or not with C20 (5 or 10 mg/kg) or with Cyclosporin (CsA, 30 mg/kg), or vehicle-treated, as indicated. IL-2 serum level in untreated WT and in PKCθ^−/−^ mice treated or not with ConA, is also shown. Statistical significance was determined by a one-way ANOVA using a Dunnett's post-test (*, p < 0,05; means ± STDEV; n = 3 for each group). The mild difference in IL-2 production observed between the two doses of C20 was not significant. B. Morphological analysis of TA muscle in WT mice treated or not treated with C20. Upper panel: immunofluorescence analysis for Laminin in TA muscle cryosections from vehicle- or C20-treated WT mice, as indicated (bar = 400 μm); Lower panel: cross sectional area (CSA) fiber distribution in TA muscle of vehicle- (black bars) or C20- (grey bars) treated WT mice; the percentage of fibers within the given range of CSA is indicated for each group of mice (n = 3 mice per group). C. Food deprivation-induced muscle atrophy in WT mice treated or not with C20. Upper panel: body weight of WT mice treated (grey bars) or not (black bars) with C20, in fed (plain bars) or fasted (dotted bars) conditions, as indicated. Lower panel: frequency distribution of the fibers CSA in TA muscle derived from WT fed (black bars) or fasted treated (grey dotted bars) or not (black dotted bars) with C20, as indicated. D. Analysis of the heart in WT mice treated or not with C20. Upper panel: Masson's Trichrome staining of crysections of heart from WT mice treated or not treated with C20, as indicated. Lower panel: Real Time PCR analysis of ANP, βMyHC and TGFβ expression level in hearts from vehicle- or C20-treated WT mice, as indicated; the level of expression in hearts from age-matched PKCθ^−/−^ mice is also shown (*, p < 0,05; means ± STDEV; n = 3 mice for each group). Gapdh level of expression was used to normalize the values. Statistical significance was determined by Student's *t*-test (*, p < 0.05; means ± STDEV; n = 5 mice per group).

In preliminary studies, we tested the ability of the PKCθ inhibitor C20 to prevent Concanavalin A (ConA)-induced T cell activation in vivo in C57BL/10ScSn WT mice. ConA is a well-known T cell mitogen and, since PKCθ is an essential factor promoting T cells activation and proliferation, its lack/inhibition should prevent ConA-induced T-cell proliferation and IL2 production. As shown in Fig. 1A, ConA treatment induced high level of serum IL-2 compared to the untreated. As expected, Cyclosporine (CsA) pre-treatment, a well-known immune-suppressor, abolished ConA-induced serum IL-2. C20 pre-treatment, at both 5 and 10 mg/kg doses, also abolished ConA-induced IL-2 production, at similar level as CsA. Lack of PKCθ in PKCθ^−/−^ mice also prevented ConA-induced IL-2 production, as it did C20 treatment.

**Supplemental Fig. S2:**
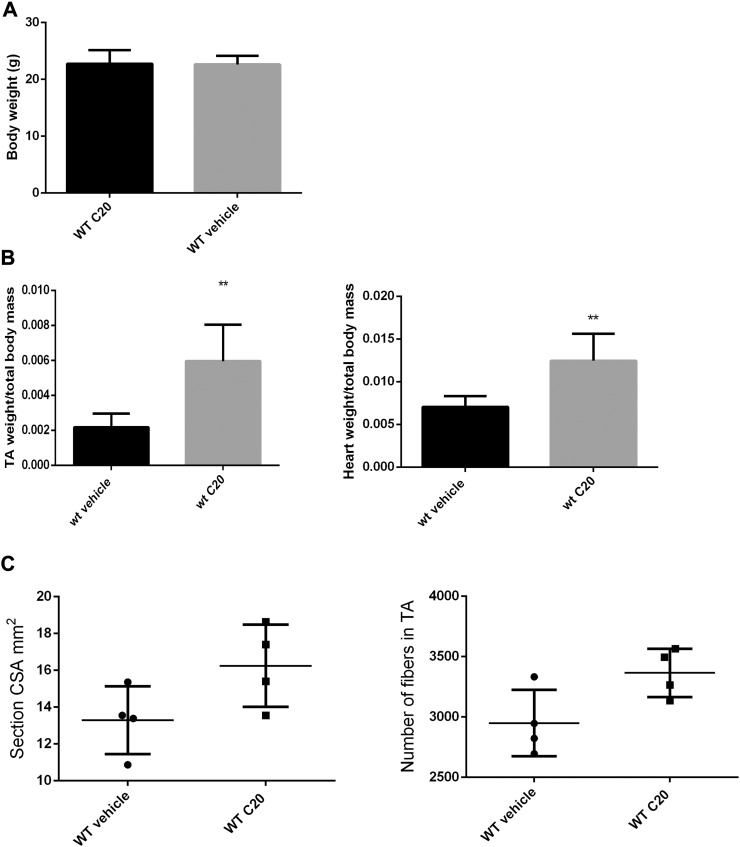
A: Body weight of WT mice, treated or not with C20; B: TA (left panel) and heart (right panel) weight from WT mice treated or not with C20, as indicated; C: TA muscle CSA (left panel) and total number of myofibers/section rom WT mice treated or not with C20. Each point represents one mouse. Black bars: WT vehicle-treated; grey bars: WT C20-treated.

Further, we investigated whether C20 treatment may results in detrimental effects in skeletal and cardiac muscle homeostasis and activity. We used 4-week-old C57BL/10ScSn WT mice for these analyses. We choose this age because it is still a reasonably young age in *mdx* to make the treatment effective, and, on the other hand, a reasonably old age to prevent eventual adverse effect in skeletal muscle growth. In fact, PKCθ expression/activity is required mostly during the early stages of skeletal muscle development, up to 4–5 weeks of age, and its lack/inhibition at these early stages may interfere with muscle growth (Madaro et al., 2011). Four-week-old WT mice were IP injected with the C20 daily for 2 weeks, after which the mice were sacrificed and weighted. TA muscles and heart were then dissected, weighted and analyzed for morphological and molecular features. No differences in body weight were detected in mice treated with C20 compared to the untreated ones (Supplemental Fig. S2). Intriguingly, an increase in TA muscle weight, normalized to body weight, was observed in C20 treated mice compared to untreated (Supplemental Fig. S2). However, myofibers CSA, measured in all the fibers of the entire section, was similar in TA derived from C20-treated mice and untreated (Fig. 1B). Moreover, C20 treatment did not alter the ability of muscle to adapt to external stimuli, such as fasting (Fig. 1C). Indeed, we previously demonstrated that lack of PKCθ, in the PKCθ^−/−^ mice, prevented fasting-induced atrophy in skeletal muscle (Madaro et al., 2013), thus we also wondered whether PKCθ acute inhibition may alter this response. Six-week-old WT mice were treated with C20 (10 mg/kg) for 2 weeks, and food was removed for the last 24 h, to induce muscle atrophy. We choose this age because it was the age of the mice used in the previous study. The mice were sacrificed and analyzed for body weight and myofiber CSA. As shown in Fig. 1C, after 24 h of starvation, C20-treated and vehicle-treated mice showed similar reduction in body weight, and similar distribution of myofiber CSA in TA muscle (Fig. 1C). Interestingly, the observed increase in TA muscle weight might be accounted for the increase in myofibers number. In fact, we found that also TA muscle CSA was increased due to increase in the number of myofibers (Supplemental Fig. S2). Together, these results demonstrate that C20 treatment does not alter limb muscle morphology and function, rather it induces increased muscle mass and myofiber number.

In addition, an increase in heart weight, normalized to body weight, was also observed in C20-treated mice compared to untreated (Supplemental Fig. S2). However, no alterations in cardiac muscle structure or deposition of collagen was observed, nor in the expression of genes involved in pathological LV remodeling or fibrosis (Fig. 1D). We previously showed that lack of PKCθ results in increased fibrosis and expression of Atrium Natriuretic Peptide (ANP), β-Myosin heavy chain (β-MyHC) and transforming growth factor β (TGFβ) in 8-week-old mice (Paoletti et al., 2010). As shown in Fig. 1D, no fibrosis was detected in heart derived from both untreated or C20-treated mice. Accordingly, the expression of ANP, β-MyHC and TGFβ was similar in hearts derived from untreated and C20 treated mice. Conversely, ANP expression was significantly induced in hearts derived from 6-week old PKCθ^−/−^ mice, as expected.

Together, these data demonstrate that C20 treatment in vivo is effective in inhibiting T cell activation, and that 2 week C20 treatment has no apparent detrimental effects on both skeletal and cardiac muscle homeostasis and maintenance.

### 3.2 C20 Treatment Prevents Damage and Inflammation in Dystrophic Muscles

**Fig. 2:**
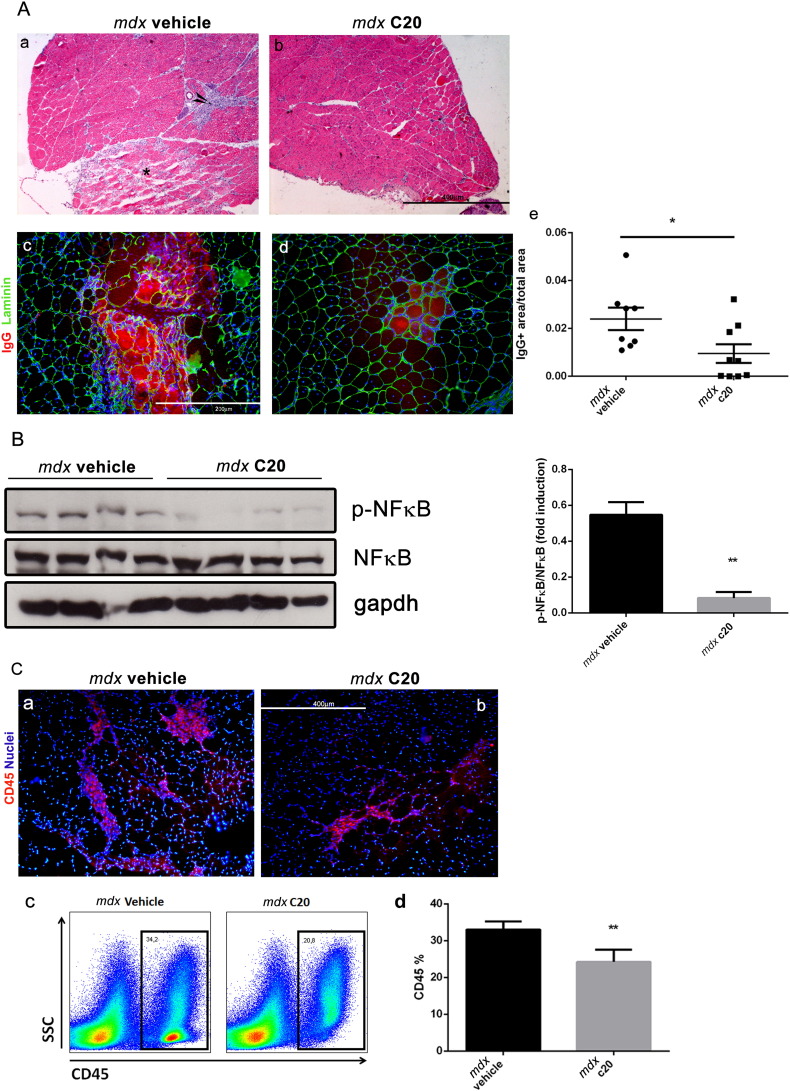
C20 treatment prevents damage and inflammation in dystrophic muscles. A. Hematoxylin/Eosin staining (a and b) of GA muscles from 6-week-old *mdx* mice treated with the C20 (b), or with the vehicle (a), the last two weeks. The asterisk indicates myonecrosis area and arrow indicates inflammatory area. Double immunofluorescence analysis of IgG and laminin (c and d) in serial cryosections as in a and b, respectively. The extend of IgG positive area over the total area of GA muscle section from each individual is shown in e. The entire muscle was used for quantification, and expressed as the average measured in three different sections/mouse; each dot represents a single mouse. (Bar = 400 μm (a and b); = 200 μm (c and d). B. Western blot analysis of NFκB (p65) expression and phosphorylation in total protein extract of GA muscles derived from vehicle and C20 treated *mdx* mice, as indicated; the ratio between pNFκB/NFκB is shown in the right as revealed by densitometric analysis. C. Representative image of CD45 (red) immunofluorescence staining of GA muscle section from vehicle (a) and C20 (b) treated *mdx* mice. Hoechst (blue) was used to visualise the nuclei. (Bar = 400 μm). (c) Representative picture of cytofluorimetric analysis of CD45^+^ cells in GA muscles derived from vehicle- (left plot) or C20- (right plot) treated *mdx* mice; (d) cytofluorimetric analysis of CD45^+^ cells in GA muscles derived from C20- (grey bar) or vehicle- (black bar) treated *mdx* mice (*p < 0,05; means ± STDEV; n = 8/9 mice/group). Statistical significance was determined by Student's *t*-test.

Next, four-week-old *mdx* mice were treated with 10 mg/kg C20 or with the vehicle for 2 weeks. After treatment, mice (6-week-old) were sacrificed and myofiber degeneration was evaluated in cryosections of GA muscle by IgG staining. As shown in Fig. 2A, significant reduction in the extension of the area including degenerating myofibers was observed in GA muscles derived from the mice treated with the C20, compared to the vehicle-treated mice (Fig. 2A).

We then verified whether the reduced muscle damage observed in C20-treated *mdx* mice might depend, at least in part, on reduced inflammatory response, since the primary cause of the disease, that is lack of dystrophin, was not removed. Western blot analysis revealed that the high level of phosphorylation/activation of the inflammation-associated factor NFκB, observed in muscle of vehicle-treated *mdx* mice, was significantly reduced in muscle of C20-treated *mdx* mice (Fig. 2B). Of note, reduced extension of the area containing infiltrated inflammatory CD45^+ ve^ cells was observed by immunofluorescence in muscle from C20-treated mice compared to vehicle-treated (Fig. 2C). FACS analysis revealed a 20% reduction of CD45^+ ve^ cells in mononucleated cells isolated from muscle of C20-treated mice compared to vehicle-treated (Fig. 2C).

### 3.3 C20 Treatment Boosts Muscle Regeneration in *mdx* Mice

**Fig. 3:**
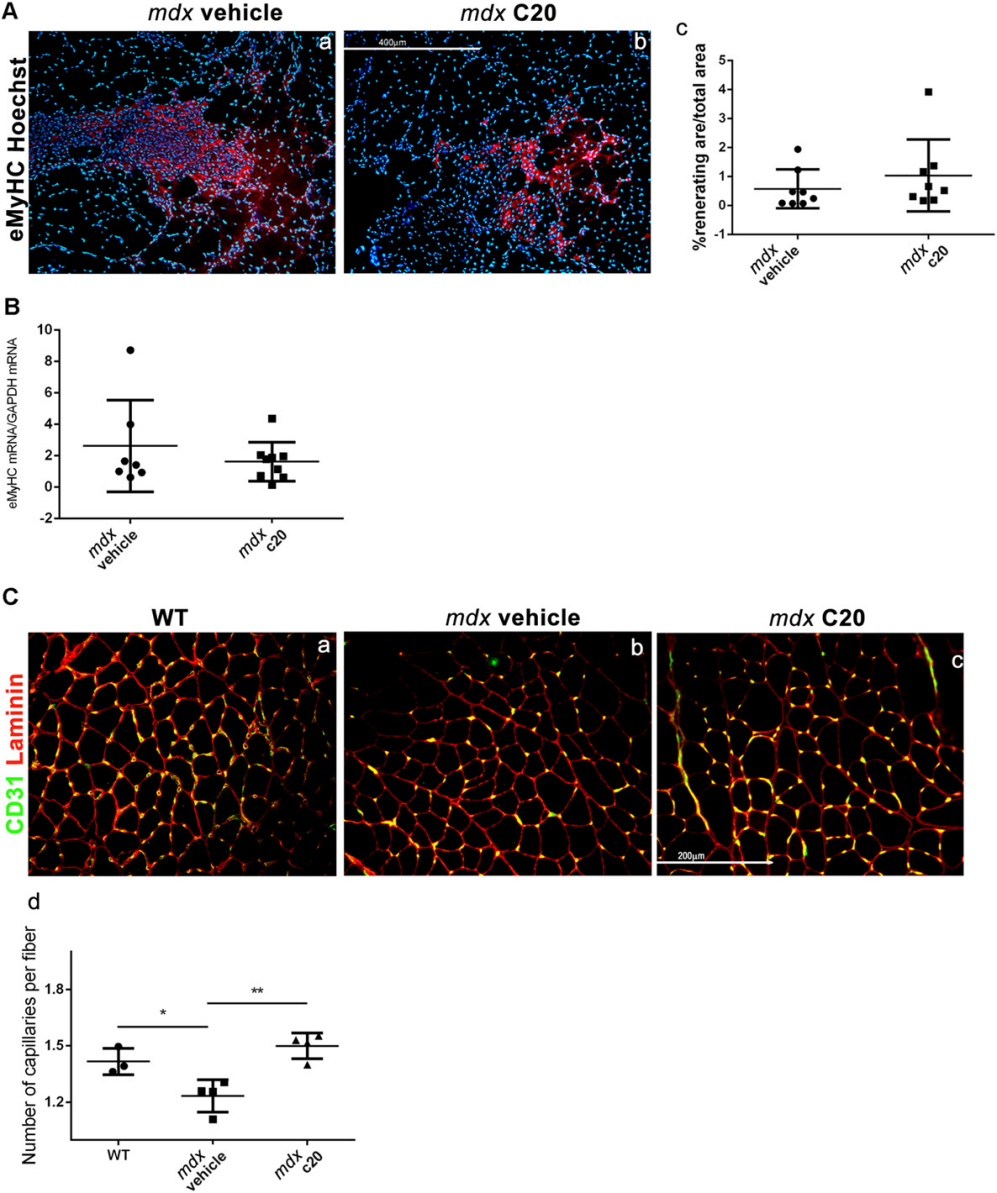
C20 treatment improves muscle regeneration in 6-week-old mdx mice. A. Immunofluorescence analysis of eMyHC (red) expression in GA muscle cryosections derived from *mdx* mice treated with the C20 (b) or with the vehicle (a). Representative images are shown. Hoechst (blue) was used to visualise the nuclei. (Bar = 400 μm). (c) Ratio of eMyHC^+ ve^ area over the total area of each section. The entire section was used for quantification, and three sections/mouse were averaged; each dot represents a single mouse. Statistical significance was determined by the Student's *t*-test. (*, p < 0,05; means ± SEM; n = 8/9 mice/group). B. Real Time PCR analysis of eMyHC level of expression in vehicle- and C20-treated *mdx* mice, as indicated. C. Immunofluorescence analysis of CD31 expression in GA muscle cryosections derived from WT (a) *mdx* mice treated with the C20 (c) or with the vehicle (b) (Bar = 200 μm). (d) The number of capillaries/fiber was determined in each mouse; at least 3 sections per muscle were used for quantification. Statistical significance was determined by a one-way ANOVA using a Dunnett's post-test. (*, p < 0,05; means ± SEM; n = 4 mice/group).

Muscle regeneration was then analyzed in GA muscles derived from *mdx* mice treated or not treated with C20, as above, by immunofluorescence for the expression of eMyHC. As shown in Fig. 3A, the extent of area including regenerating myofibers was similar in muscle derived from C20 treated *mdx* mice, compared to the vehicle-treated. qRT-PCR analysis confirmed that the level of expression of eMyHC was similar in muscle derived from C20-treated mdx mice and untreated (Fig. 3B).

**Supplemental Fig. S3:**
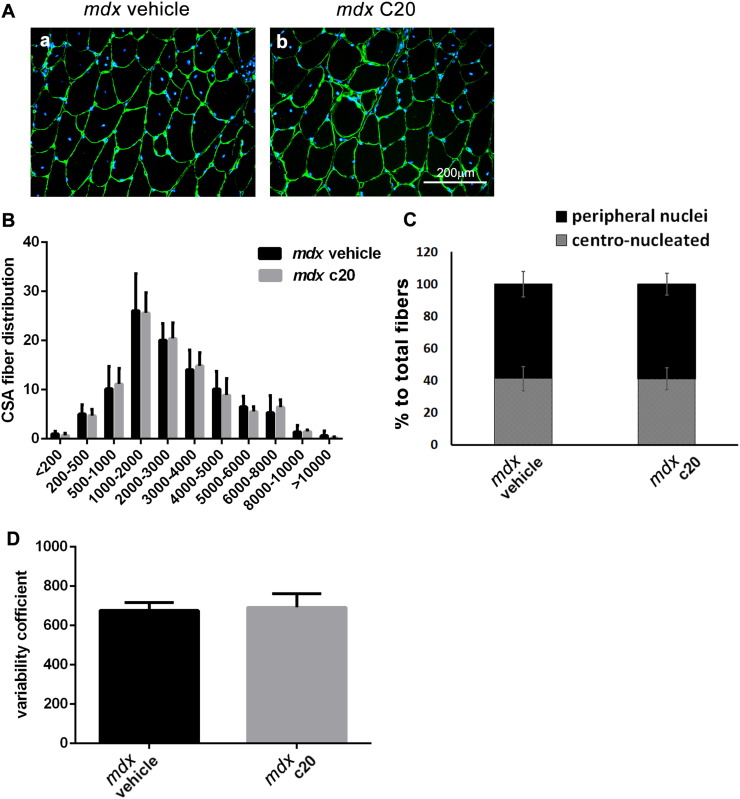
A. Laminin staining of TA muscles from 6-week-old WT mice treated with the vehicle (a) or with the C20 (b) for 2 weeks. B. CSA fiber distribution in TA muscles from 6-week-old WT mice treated with the vehicle or with the C20 for 2 weeks. C. Percentage of centro-nucleated and peripheral-nucleated fibers in TA muscle from 6-week-old WT mice treated with the vehicle or with the C20. D. Myofiber variability coefficient in TA muscles from 6-week-old WT mice treated or not with the C20. (means ± STDEV; n = 3 for each group).

Since we observed less damage in C20-treated muscle than in vehicle-treated (Fig. 2), but muscle regeneration was similar, this fact implies that the ratio between the damaged and the regenerated area is lower in C20-treated muscle compared to vehicle-treated, suggesting that muscle regeneration is boosted by C20 treatment. However, C20 treatment does not modify the myofibers CSA, nor variability coefficient or the ratio of centro-nucleated vs peripheral nucleated myofibers (Supplemental Fig. S3).

Since reduced capillarity in muscles from *mdx* mice was previously described as a result of necrosis-induced muscle ischemia (Palladino et al., 2013), we then counted the number of capillaries in GA muscles from C20-treated and untreated *mdx* mice. In line with the observation of boosted regeneration, the number of capillaries was higher in muscle derived from C20-treated than in muscle derived from vehicle-treated *mdx* mice, and was similar to that counted in GA muscles from age-matched WT mice (Fig. 3C).

### 3.4 C20 Treatment Prevents the Drop-in Force Observed in Muscle From *mdx* Mice Ex vivo

**Fig. 4:**
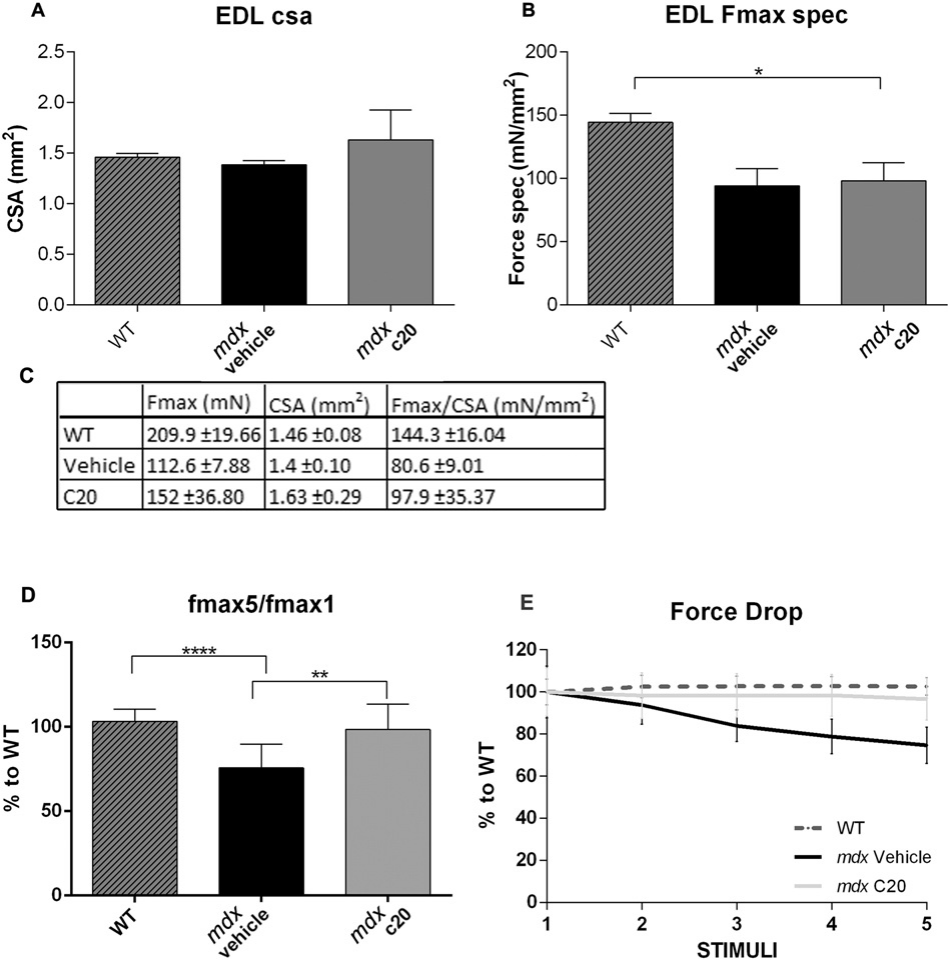
C20 treatment prevents the drop-in force observed in muscle from *mdx* mice ex vivo. A. CSA of EDL muscles derived from 6-week old WT, vehicle- or C20-treated *mdx* mice; B. Specific Force, generated by of EDL muscles as in A, obtained as the ratio of the Fmax generated following the first stimulation/CSA; D. Table including: the maximum force (Fmax) measured from the first tetanic stimulation of EDL muscles from WT mice and *mdx* mice treated with the vehicle and with C20, as indicated; the muscle cross sectional area (CSA) for each muscle and the ratio between them (Fmax/CSA). (*p < 0,05 was obtained comparing Fmax/CSA from WT to both vehicle- or C20-treated *mdx*; means ± STDEV; n = 5 for each group). D. The drop-in force, evaluated as the value of force developed during the last stimulation (F5) divided for the initial value of force (F1), expressed as percentage: F5/F1%. (*p < 0,05, means ± STDEV; n = 5 for each group). E. Force developed by EDL muscles from WT (dotted line) and *mdx* mice treated with the C20 (grey line) or with the vehicle (black line), during repeated stimulations, as indicated. The value determined in each stimulation is expressed as the percentage to the value obtained from each group at the first stimulation, and assumed as 100. Statistical significance was determined by Two Way ANOVA test with Tukey's multiple comparisons (*p < 0,05, means ± STDEV; n = 5 for each group).

We then verified whether C20 treatment may improve dystrophic muscle contraction activity. In fact, although lack of dystrophin primarily leads to muscle contraction defects, inflammation and fibrosis contribute to worsen the muscle weakness in dystrophic mice and in DMD patients. EDL muscle was dissected from WT, vehicle- or C20-treated *mdx* mice and analyzed ex vivo for contractile parameters. As expected, a significant reduction of the Fmax generated from muscle derived from vehicle-treated *mdx* mice was observed compared to WT. C20 treatment partially rescued the observed reduction in terms of absolute generated Fmax (Fig. 4A). However, in line with what observed in TA muscle, we found that muscle CSA was higher in EDL derived from C20-treated *mdx* mice compared to *mdx*; thus, the specific force (Fmax/CSA) generated remained unaltered (Fig. 4A). Conversely, a significant reduction in force drop of muscle derived from C20-treated *mdx* mice, compared to untreated *mdx*, was found (Fig. 4B). Notably, the force produced by EDL muscle derived from untreated *mdx* mice decreased after each stimulation, presumably because of muscle damage, starting from the third stimulation., After 5 consecutive stimulations, the force generated by EDL derived from untreated *mdx* muscles (Fmax5) was reduced of about 25% compared to the initial one (Fmax1). By contrast, the force produced by EDL derived from C20-treated *mdx* mice was maintained almost unaltered between the first and the last stimulation, similarly to what observed in muscle derived from WT mice (Fig. 4).

### 3.5 C20 Treatment Ameliorates Fatigue Resistance in *mdx* Mice

**Fig. 5:**
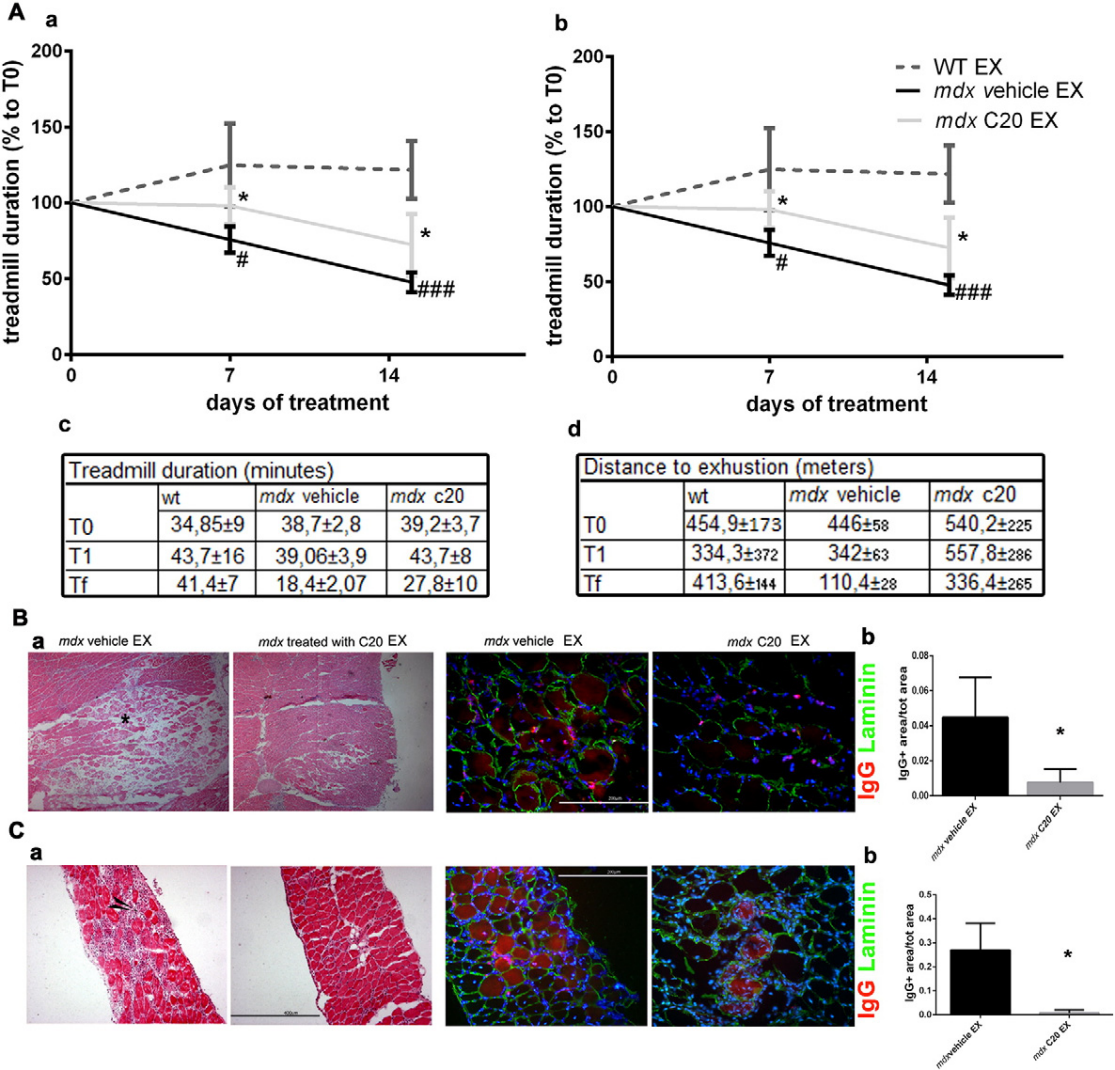
C20 ameliorates fatigue resistant and protects muscle from degeneration after a treadmill assay. A. Exhaustion Treadmill test performed on vehicle (black line) and C20 (grey line) treated *mdx* mice; WT mice (dotted line) were also used for comparison (n = 5 each group). Muscle strength was measured as duration (a) or distance (b) to exhaustion, measured 7 (T1) and 14 (Tf) days after the beginning of the treatment; the obtained values are expressed as percentage in respect to the values measured at the beginning of the treatment (T0). B. The values of treadmill duration (a) and distance (b) of WT and *mdx* treated with the vehicle and with the C20 at different time points during the treatment are reported in tables c and d, respectively. C. Representative images of Hematoxylin/Eosin (a and b) or of the IgG and Laminin double immunofluorescence analysis (c and d) of GA muscle cryosections derived from C20- or vehicle-treated *mdx* mice, as indicated, and subjected to treadmill exercise. GA muscles were harvested at the end of the treadmill test, (Tf), as in A. (Bar = 400 μm (a and b); = 200 μm (c and d)). The asterisk indicates myonecrosis area and arrow indicates inflammatory area. The ratio between the area positive for IgG (as sign of necrosis) over the total area of muscle section from exercised C20- or vehicle-treated *mdx* mice is shown in e. At least 3 sections per muscle were used for quantification. Statistical significance was determined by Student's *t*-test. D. Same as in **C**, but in DIA muscle from the same mice (means ± STDEV; n = 5 for each group). *Significantly different (p < 0.05) from vehicle and # significantly different from WT.

Next, we evaluated performance of C20- or vehicle-treated *mdx* mice in a fatigue resistance protocol on a treadmill, during the treatment. A group of WT mice was also included. Distance to exhaustion and time to exhaustion were measured for each mouse at day 7 and 14 during C20 treatment, and was compared to the day 0 values. As shown in Fig. 5A, the initial treadmill duration, at T0, was similar in all group of mice (WT, vehicle-, or C20-treated *mdx*). The initial distance, at T0, run by WT and vehicle-treated *mdx* mice was also similar, while the one run by the C20-treated *mdx* mice appeared greater, although this difference was not significant. Both time to exhaustion and distance, run by WT mice, at day 7 (T1) and 14 (Tf) remained unaltered. By contrast, both duration and distance was significantly reduced in vehicle-treated *mdx* mice at T1, and further at Tf: they covered on average a shorter distance and became exhausted much faster, compared to WT (Fig. 5A). Interestingly, C20-treated *mdx* mice covered on average a longer distance in comparison to their untreated counterparts, already at T1, as well as at Tf. Also, C20-treated *mdx* mice became exhausted much slower at both T1 and T2, compared to vehicle-treated *mdx* mice (Fig. 5A)

Since susceptibility to the exhaustion training in *mdx* mice might be primarily due to exercise-induced muscle damage, due to the absence to dystrophin, we next evaluated morphology of muscle derived from exercised mice. As shown by H/E and IgG staining, both GA and DIA muscles derived from exercised *mdx* mice were dramatically damaged, and many IgG stained necrotic fibers were observed. By contrast, C20 treatment preserved muscle integrity after training, in both GA and DIA, counteracting the muscle damage observed in untreated *mdx* mice (Fig. 5B). Together, these results demonstrate that acute inhibition of PKCθ is very efficient in recovering muscle performance in *mdx*, by preserving muscle integrity.

## 4 Discussion

This study demonstrates that pharmacological inhibition of PKCθ in 4-week-old *mdx* mice, significantly prevents muscle wasting and inflammatory response, while maintains muscle regeneration and improves function and performance. We used the C20, one of the previously identified potent and selective PKCθ inhibitors (Cywin et al., 2007), and show that in vivo C20 treatment prevented ConA-induced T cells activation, as expected for a PKCθ inhibitor (Bermejo et al., 2015, Cywin et al., 2007, Zanin-Zhorov et al., 2010). Although its eventual clinical application should be further investigated, our data provide proof of principle that pharmacological targeting of PKCθ in muscular dystrophy would be effective in ameliorating the disease, as our previous genetic studies suggested. We show that two week C20 treatment of 4-week-old *mdx* mice significantly reduced histo-pathological features of dystrophic muscle. We decided to treat *mdx* mice at 4 week of age, when immune cells are infiltrating muscle, thus, if our hypothesis was correct, we would expect that C20 treatment should prevent/reduce inflammatory response. On the other hand, previous studies have demonstrated that PKCθ expression/activity is required for complete histogenesis, differentiation and homeostasis of skeletal muscle, as well as for cardiomyocyte survival and remodeling (Camerino et al., 2014, Madaro et al., 2011, Messina et al., 2010, Paoletti et al., 2010, Tokugawa et al., 2009, Zappelli et al., 1996). We already showed that in a context of chronic inflammation, where immune cells activity is a key determinant, as in muscular dystrophy, lack of PKCθ predominantly affects immune response ameliorating the disease, and no obvious adverse effects were observed in other tissues (i.e. skeletal and cardiac muscle) (Madaro et al., 2012). We show here that C20 treatment of 4-week-old WT mice, did not result in deleterious effects on either skeletal or cardiac muscle phenotype, nor on the ability of skeletal muscle to respond to external stimuli, as to fasting; however, increased skeletal and cardiac muscle mass was observed. In TA muscle this is possibly due to a rise in the number of myofibers, as we observed, which might be beneficial in a context of muscle wasting; although the underlying mechanism(s) is not clear yet, it may explain the boosted muscle regenerative ability, observed in C20-treated dystrophic mice. In fact, we did not observe myofiber number increase in TA derived from C20-treated *mdx* mice, and the percentage of centrally and peripherally nucleated myofiber was unaltered. However, since myofiber damage was reduced, compared to muscle from untreated *mdx* mice, as discussed below, the maintenance of muscle regeneration might be due to increase in satellite cells activation and myogenesis, in line with the C20-induced fiber number increase in WT muscle. Besides, we showed that 2 week C20 treatment of 4-week-old *mdx* mice, significantly reduced muscle fiber degeneration, immune cells infiltration, as well as inflammatory pathways activation, such as NFκB. Indeed, NFκB is activated very early in muscle from *mdx* mice, probably because of contraction-induced muscle injury. NFκB is known to regulate the expression of many inflammatory genes, including cytokines and chemokines in both immune cells and muscle fibers, thus maintaining an inflammatory environment.

Interestingly, despite the reduced muscle damage, the level of muscle regeneration was similar in C20-treated and untreated *mdx* mice, as shown by the extent of the area including eMyHC expressing fibers. It is generally accepted that the level of muscle regeneration should be proportional to the level of damage, since it is activated to replace damaged fibers. However, in DMD muscle progenitor cells activation/differentiation is limited, mainly due to the exhaustion of muscle progenitor cells pool, given the continuous cycles of degeneration/regeneration, but also chronic inflammation contributes to make an unfavorable environment for their activation/differentiation. The fact that C20 treatment, as well as PKCθ genetic ablation (Madaro et al., 2012) reduces muscle damage but maintains muscle regeneration in *mdx*, suggests that the treatment makes a more favorable environment for muscle precursors cells to differentiate by reducing inflammation and thus allowing muscle repair, a possibility which is being further investigated. Moreover, as suggested here, C20 treatment may increase myofibers number in WT muscle, ability that may contribute to the maintenance of muscle regeneration in dystrophic muscle.

Importantly, C20 treatment significantly improved muscle functionality both ex vivo and in vivo, and preserved muscle from contraction-induced muscle damage. We showed that although the force generated from EDL muscle derived from C20-treated *mdx* mice was higher than that from untreated, the specific force was similar. However, a significant reduction in force drop of muscle derived from C20-treated *mdx* mice, compared to untreated *mdx* was observed. This fact might imply that muscle integrity is maintained by C20 treatment, making it more resistant to the subsequent contractions, as observed following the exhaustion treadmill assay. On the other hand, the eventual improved blood supply might also prevent loss of force, thus contributing to resistance. Accordingly, C20-treated *mdx* mice performed much better than untreated *mdx* littermates in an exhaustion treadmill assay, probably because of the maintenance of muscle integrity. In fact, we show that the expected muscle damage observed in exercised *mdx* mice was significantly prevented by C20 treatment. These results were quite surprising since dystrophin is lacking in C20-treated mice, as well. It is conceivable that C20 treatment, reducing the inflammatory response, created a qualitative environment that makes the dystrophic muscle more resistant to the damage exerted by mechanical contraction, as it was observed also when *mdx* mice were treated with anti-IL6 antibody (Pelosi et al., 2015)

Our findings make PKCθ a very attractive target to counteract Duchenne Muscular Dystrophy and provide proof of principle that its pharmacological inhibition in DMD can be proposed in order to counteract the disease, as long as specific and selective PKCθ inhibitors, with a defined therapeutic profile will be characterized. Moreover, the eventual effects of long term PKCθ inhibition should also be investigated in the future. Although the possibility that C20 may act also on other targets, together with PKCθ, cannot be ruled out, it is noteworthy that C20 treatment in *mdx* mostly resembles the phenotype observed when PKCθ was ablated in *mdx* mice. Differently from the activity of other anti-inflammatory agents, given the peculiar, specific, activity of PKCθ on T cell, its inhibition should result in an immune-modulation rather than a general immune suppression, making PKCθ a potential target to reduce the immune response avoiding eventual side effects associated with the drugs currently used in clinic.

The following are the supplementary data related to this article.

## Authors Contributions

VM, AB and LM performed histological analyses; VM performed treadmill functional analyses and statistical analyses; VM and PF managed the mouse model; PF performed molecular analyses; BLO performed cytofluorimetric analyses; SP performed the ex-vivo experiments; SP, ER and AM designed and organized the ex vivo experiments; MB, LM and VM designed the study; MB and VM organized the experiments and wrote the paper. All authors contributed to acquisition, analysis, and interpretation of data.

## References

[bb0005] Acharyya S., Villalta S.A., Bakkar N., Bupha-Intr T., Janssen P.M., Carathers M., Li Z.W., Beg A.A., Ghosh S., Sahenk Z. (2007). Interplay of IKK/NF-kappaB signaling in macrophages and myofibers promotes muscle degeneration in Duchenne muscular dystrophy. J. Clin. Invest..

[bb0010] Anderson K., Fitzgerald M., Dupont M., Wang T., Paz N., Dorsch M., Healy A., Xu Y., Ocain T., Schopf L. (2006). Mice deficient in PKC theta demonstrate impaired in vivo T cell activation and protection from T cell-mediated inflammatory diseases. Autoimmunity.

[bb0015] Angelini C. (2007). The role of corticosteroids in muscular dystrophy: a critical appraisal. Muscle Nerve.

[bb0020] Angelini C., Peterle E. (2012). Old and new therapeutic developments in steroid treatment in Duchenne muscular dystrophy. Acta Myol..

[bb0025] Bengtsson N.E., Seto J.T., Hall J.K., Chamberlain J.S., Odom G.L. (2016). Progress and prospects of gene therapy clinical trials for the muscular dystrophies. Hum. Mol. Genet..

[bb0030] Bermejo M., Lopez-Huertas M.R., Hedgpeth J., Mateos E., Rodriguez-Mora S., Maleno M.J., Plana M., Swindle J., Alcami J., Coiras M. (2015). Analysis of protein kinase C theta inhibitors for the control of HIV-1 replication in human CD4 + T cells reveals an effect on retrotranscription in addition to viral transcription. Biochem. Pharmacol..

[bb0035] Boschelli D.H., Subrath J., Niu C., Wu B., Wang Y., Lee J., Brennan A., Ho M., Deng B., Yang X. (2010). Optimization of 5-vinylaryl-3-pyridinecarbonitriles as PKCtheta inhibitors. Bioorg. Med. Chem. Lett..

[bb0040] Camerino G.M., Bouche M., De Bellis M., Cannone M., Liantonio A., Musaraj K., Romano R., Smeriglio P., Madaro L., Giustino A. (2014). Protein kinase C theta (PKCtheta) modulates the ClC-1 chloride channel activity and skeletal muscle phenotype: a biophysical and gene expression study in mouse models lacking the PKCtheta. Pflugers Arch..

[bb0045] Chand S., Mehta N., Bahia M.S., Dixit A., Silakari O. (2012). Protein kinase C-theta inhibitors: a novel therapy for inflammatory disorders. Curr. Pharm. Des..

[bb0050] Cossu G., Previtali S.C., Napolitano S., Cicalese M.P., Tedesco F.S., Nicastro F., Noviello M., Roostalu U., Natali Sora M.G., Scarlato M. (2015). Intra-arterial transplantation of HLA-matched donor mesoangioblasts in Duchenne muscular dystrophy. EMBO Mol. Med..

[bb0055] Curnock A., Bolton C., Chiu P., Doyle E., Fraysse D., Hesse M., Jones J., Weber P., Jimenez J.M. (2014). Selective protein kinase Ctheta (PKCtheta) inhibitors for the treatment of autoimmune diseases. Biochem. Soc. Trans..

[bb0060] Cywin C.L., Dahmann G., Prokopowicz A.S., Young E.R., Magolda R.L., Cardozo M.G., Cogan D.A., Disalvo D., Ginn J.D., Kashem M.A. (2007). Discovery of potent and selective PKC-theta inhibitors. Bioorg. Med. Chem. Lett..

[bb0065] Del Prete Z., Musaro A., Rizzuto E. (2008). Measuring mechanical properties, including isotonic fatigue, of fast and slow MLC/mIgf-1 transgenic skeletal muscle. Ann. Biomed. Eng..

[bb0070] Evans N.P., Misyak S.A., Robertson J.L., Bassaganya-Riera J., Grange R.W. (2009). Dysregulated intracellular signaling and inflammatory gene expression during initial disease onset in Duchenne muscular dystrophy. Am. J. Phys. Med. Rehabil..

[bb0075] Evans N.P., Misyak S.A., Robertson J.L., Bassaganya-Riera J., Grange R.W. (2009). Immune-mediated mechanisms potentially regulate the disease time-course of Duchenne muscular dystrophy and provide targets for therapeutic intervention. PM R.

[bb0080] Evans N.P., Call J.A., Bassaganya-Riera J., Robertson J.L., Grange R.W. (2010). Green tea extract decreases muscle pathology and NF-kappaB immunostaining in regenerating muscle fibers of mdx mice. Clin. Nutr..

[bb0085] Fang X., Wang R., Ma J., Ding Y., Shang W., Sun Z. (2012). Ameliorated ConA-induced hepatitis in the absence of PKC-theta. PLoS One.

[bb0090] George D.M., Breinlinger E.C., Friedman M., Zhang Y., Wang J., Argiriadi M., Bansal-Pakala P., Barth M., Duignan D.B., Honore P. (2015). Discovery of selective and orally bioavailable protein kinase Ctheta (PKCtheta) inhibitors from a fragment hit. J. Med. Chem..

[bb0095] Giannoni F., Lyon A.B., Wareing M.D., Dias P.B., Sarawar S.R. (2005). Protein kinase C theta is not essential for T-cell-mediated clearance of murine gammaherpesvirus 68. J. Virol..

[bb0100] Gupta S., Manicassamy S., Vasu C., Kumar A., Shang W., Sun Z. (2008). Differential requirement of PKC-theta in the development and function of natural regulatory T cells. Mol. Immunol..

[bb0105] Hage-Sleiman R., Hamze A.B., Reslan L., Kobeissy H., Dbaibo G. (2015). The novel PKCtheta from benchtop to clinic. J. Immunol. Res..

[bb0110] Heier C.R., Damsker J.M., Yu Q., Dillingham B.C., Huynh T., Van der Meulen J.H., Sali A., Miller B.K., Phadke A., Scheffer L. (2013). VBP15, a novel anti-inflammatory and membrane-stabilizer, improves muscular dystrophy without side effects. EMBO Mol. Med..

[bb0115] Jimenez J.M., Boyall D., Brenchley G., Collier P.N., Davis C.J., Fraysse D., Keily S.B., Henderson J., Miller A., Pierard F. (2013). Design and optimization of selective protein kinase C theta (PKCtheta) inhibitors for the treatment of autoimmune diseases. J. Med. Chem..

[bb0120] Katoh T., Takai T., Yukawa T., Tsukamoto T., Watanabe E., Mototani H., Arita T., Hayashi H., Nakagawa H., Klein M.G. (2016). Discovery and optimization of 1,7-disubstituted-2,2-dimethyl-2,3-dihydroquinazolin-4(1H)-ones as potent and selective PKCtheta inhibitors. Bioorg. Med. Chem..

[bb0125] Kharraz Y., Guerra J., Pessina P., Serrano A.L., Munoz-Canoves P. (2014). Understanding the process of fibrosis in Duchenne muscular dystrophy. Biomed. Res. Int..

[bb0130] Kumar A., Boriek A.M. (2003). Mechanical stress activates the nuclear factor-kappaB pathway in skeletal muscle fibers: a possible role in Duchenne muscular dystrophy. FASEB J..

[bb0135] Kumar A., Khandelwal N., Malya R., Reid M.B., Boriek A.M. (2004). Loss of dystrophin causes aberrant mechanotransduction in skeletal muscle fibers. FASEB J..

[bb0140] Kwon M.J., Ma J., Ding Y., Wang R., Sun Z. (2012). Protein kinase C-theta promotes Th17 differentiation via upregulation of Stat3. J. Immunol..

[bb0145] Ma J., Ding Y., Fang X., Wang R., Sun Z. (2012). Protein kinase C-theta inhibits inducible regulatory T cell differentiation via an AKT-Foxo1/3a-dependent pathway. J. Immunol..

[bb0150] Madaro L., Bouche M. (2014). From innate to adaptive immune response in muscular dystrophies and skeletal muscle regeneration: the role of lymphocytes. Biomed. Res. Int..

[bb0155] Madaro L., Marrocco V., Fiore P., Aulino P., Smeriglio P., Adamo S., Molinaro M., Bouche M. (2011). PKC{theta} signaling is required for myoblast fusion by regulating the expression of caveolin-3 and {beta}1D integrin upstream focal adhesion kinase. Mol. Biol. Cell.

[bb0160] Madaro L., Pelle A., Nicoletti C., Crupi A.M.V.B.G., Soddu S., Bouche M. (2012). PKC Theta ablation improves healing in a mouse model of muscular dystrophy. PLoS One.

[bb0165] Madaro L., Marrocco V., Carnio S., Sandri M., Bouche M. (2013). Intracellular signaling in ER stress-induced autophagy in skeletal muscle cells. FASEB J..

[bb0170] Marrocco V., Fiore P., Madaro L., Crupi A., Lozanoska-Ochser B., Bouche M. (2014). Targeting PKCtheta in skeletal muscle and muscle diseases: good or bad?. Biochem. Soc. Trans..

[bb0175] Mercuri E., Muntoni F. (2013). Muscular dystrophies. Lancet.

[bb0180] Messina S., Bitto A., Aguennouz M., Minutoli L., Monici M.C., Altavilla D., Squadrito F., Vita G. (2006). Nuclear factor kappa-B blockade reduces skeletal muscle degeneration and enhances muscle function in Mdx mice. Exp. Neurol..

[bb0185] Messina S., Bitto A., Aguennouz M., Mazzeo A., Migliorato A., Polito F., Irrera N., Altavilla D., Vita G.L., Russo M. (2009). Flavocoxid counteracts muscle necrosis and improves functional properties in mdx mice: a comparison study with methylprednisolone. Exp. Neurol..

[bb0190] Messina G., Biressi S., Monteverde S., Magli A., Cassano M., Perani L., Roncaglia E., Tagliafico E., Starnes L., Campbell C.E. (2010). Nfix regulates fetal-specific transcription in developing skeletal muscle. Cell.

[bb0195] Muntoni F. (2003). Cardiomyopathy in muscular dystrophies. Curr. Opin. Neurol..

[bb0200] Muntoni F., Torelli S., Ferlini A. (2003). Dystrophin and mutations: one gene, several proteins, multiple phenotypes. Lancet Neurol..

[bb0205] Nath P.R., Isakov N. (2014). PKCtheta-regulated signalling in health and disease. Biochem. Soc. Trans..

[bb0210] Odom G.L., Gregorevic P., Allen J.M., Chamberlain J.S. (2011). Gene therapy of mdx mice with large truncated dystrophins generated by recombination using rAAV6. Mol. Ther..

[bb0215] Palladino M., Gatto I., Neri V., Straino S., Smith R.C., Silver M., Gaetani E., Marcantoni M., Giarretta I., Stigliano E. (2013). Angiogenic impairment of the vascular endothelium: a novel mechanism and potential therapeutic target in muscular dystrophy. Arterioscler. Thromb. Vasc. Biol..

[bb0220] Paoletti R., Maffei A., Madaro L., Notte A., Stanganello E., Cifelli G., Carullo P., Molinaro M., Lembo G., Bouche M. (2010). Protein kinase Ctheta is required for cardiomyocyte survival and cardiac remodeling. Cell Death Dis..

[bb0225] Pelosi L., Giacinti C., Nardis C., Borsellino G., Rizzuto E., Nicoletti C., Wannenes F., Battistini L., Rosenthal N., Molinaro M. (2007). Local expression of IGF-1 accelerates muscle regeneration by rapidly modulating inflammatory cytokines and chemokines. FASEB J..

[bb0230] Pelosi L., Berardinelli M.G., De Pasquale L., Nicoletti C., D'amico A., Carvello F., Moneta G.M., Catizone A., Bertini E., De B.F. (2015). Functional and morphological improvement of dystrophic muscle by interleukin 6 receptor blockade. EBioMedicine.

[bb0235] Pessina P., Kharraz Y., Jardi M., Fukada S., Serrano A.L., Perdiguero E., Munoz-Canoves P. (2015). Fibrogenic cell plasticity blunts tissue regeneration and aggravates muscular dystrophy. Stem Cell Rep..

[bb0240] Pfeifhofer C., Kofler K., Gruber T., Tabrizi N.G., Lutz C., Maly K., Leitges M., Baier G. (2003). Protein kinase C theta affects Ca2 + mobilization and NFAT cell activation in primary mouse T cells. J. Exp. Med..

[bb0245] Rosenberg A.S., Puig M., Nagaraju K., Hoffman E.P., Villalta S.A., Rao V.A., Wakefield L.M., Woodcock J. (2015). Immune-mediated pathology in Duchenne muscular dystrophy. Sci. Transl. Med..

[bb0250] Sampaolesi M., Torrente Y., Innocenzi A., Tonlorenzi R., D'Antona G., Pellegrino M.A., Barresi R., Bresolin N., De Angelis M.G., Campbell K.P. (2003). Cell therapy of alpha-sarcoglycan null dystrophic mice through intra-arterial delivery of mesoangioblasts. Science.

[bb0255] Sampaolesi M., Blot S., D'Antona G., Granger N., Tonlorenzi R., Innocenzi A., Mognol P., Thibaud J.L., Galvez B.G., Barthelemy I. (2006). Mesoangioblast stem cells ameliorate muscle function in dystrophic dogs. Nature.

[bb0260] Schacke H., Docke W.D., Asadullah K. (2002). Mechanisms involved in the side effects of glucocorticoids. Pharmacol. Ther..

[bb0265] Schoepe S., Schacke H., May E., Asadullah K. (2006). Glucocorticoid therapy-induced skin atrophy. Exp. Dermatol..

[bb0270] Seto J.T., Ramos J.N., Muir L., Chamberlain J.S., Odom G.L. (2012). Gene replacement therapies for Duchenne muscular dystrophy using adeno-associated viral vectors. Curr. Gene Ther..

[bb0275] Sims T.N., Soos T.J., Xenias H.S., Dubin-Thaler B., Hofman J.M., Waite J.C., Cameron T.O., Thomas V.K., Varma R., Wiggins C.H. (2007). Opposing effects of PKCtheta and WASp on symmetry breaking and relocation of the immunological synapse. Cell.

[bb0280] Sun Z. (2012). Intervention of PKC-theta as an immunosuppressive regimen. Front. Immunol..

[bb0285] Tan S.L., Zhao J., Bi C., Chen X.C., Hepburn D.L., Wang J., Sedgwick J.D., Chintalacharuvu S.R., Na S. (2006). Resistance to experimental autoimmune encephalomyelitis and impaired IL-17 production in protein kinase C theta-deficient mice. J. Immunol..

[bb0290] Thuille N., Wachowicz K., Hermann-Kleiter N., Kaminski S., Fresser F., Lutz-Nicoladoni C., Leitges M., Thome M., Massoumi R., Baier G. (2013). PKCtheta/beta and CYLD are antagonistic partners in the NFkappaB and NFAT transactivation pathways in primary mouse CD3 + T lymphocytes. PLoS One.

[bb0295] Tokugawa S., Sakuma K., Fujiwara H., Hirata M., Oda R., Morisaki S., Yasuhara M., Kubo T. (2009). The expression pattern of PKCtheta in satellite cells of normal and regenerating muscle in the rat. Neuropathology.

[bb0300] Villalta S.A., Rosenberg A.S., Bluestone J.A. (2015). The immune system in Duchenne muscular dystrophy: friend or foe. Rare Dis..

[bb0305] Wachowicz K., Baier G. (2014). Protein kinase Ctheta: the pleiotropic T-cell signalling intermediate. Biochem. Soc. Trans..

[bb0310] Wachowicz K., Hermann-Kleiter N., Meisel M., Siegmund K., Thuille N., Baier G. (2014). Protein kinase C theta regulates the phenotype of murine CD4 + Th17 cells. PLoS One.

[bb0315] Wang L., Xiang Z., Ma L.L., Chen Z., Gao X., Sun Z., Williams P., Chari R.S., Yin D.P. (2009). Deficiency of protein kinase C-theta facilitates tolerance induction. Transplantation.

[bb0320] Zanin-Zhorov A., Ding Y., Kumari S., Attur M., Hippen K.L., Brown M., Blazar B.R., Abramson S.B., Lafaille J.J., Dustin M.L. (2010). Protein kinase C-theta mediates negative feedback on regulatory T cell function. Science.

[bb0325] Zappelli F., Willems D., Osada S., Ohno S., Wetsel W.C., Molinaro M., Cossu G., Bouche M. (1996). The inhibition of differentiation caused by TGFbeta in fetal myoblasts is dependent upon selective expression of PKCtheta: a possible molecular basis for myoblast diversification during limb histogenesis. Dev. Biol..

